# Ohmyungsamycin promotes M1-like inflammatory responses to enhance host defence against *Mycobacteroides abscessus* infections

**DOI:** 10.1080/21505594.2022.2138009

**Published:** 2022-11-15

**Authors:** Sang Min Jeon, Young Jae Kim, Thanh Quang Nguyen, Jinsheng Cui, Bui Thi Bich Hanh, Prashanta Silwal, Jin Kyung Kim, Jin-Man Kim, Dong-Chan Oh, Jichan Jang, Eun-Kyeong Jo

**Affiliations:** a Department of Microbiology, Chungnam National University School of Medicine, Daejeon, South Korea; b Infection Control Convergence Research Center, Chungnam National University School of Medicine, Daejeon, South Korea; c Department of Medical Science, Chungnam National University School of Medicine, Daejeon, South Korea; d Brain Korea 21 FOUR Project for Medical Science, Chungnam National University School of Medicine, Daejeon, South Korea; e Division of Life Science, Department of Bio & Medical Big Data (BK21 Four Program), Research Institute of Life Science, Gyeongsang National University, Jinju, South Korea; f Natural Products Research Institute, College of Pharmacy, Seoul National University, Seoul, South Korea; g Division of Applied Life Science (BK21 Four Program), Research Institute of Life Science, Gyeongsang National University, Jinju, South Korea; h Department of Microbiology, Keimyung University School of Medicine, Daegu, South Korea; i Department of Pathology, Chungnam National University School of Medicine, Daejeon, South Korea

**Keywords:** Ohmyungsamycins, *Mycobacteroides abscessus*, M1 macrophage responses, mitochondrial reactive oxygen species, nitric oxide, innate immunity

## Abstract

Ohmyungsamycin A (OMS) is a newly identified cyclic peptide that exerts antimicrobial effects against *Mycobacterium tuberculosis*. However, its role in nontuberculous mycobacteria (NTMs) infections has not been clarified. *Mycobacteroides abscessus* (Mabc) is a rapidly growing NTM that has emerged as a human pathogen in both immunocompetent and immunosuppressed individuals. In this study, we demonstrated that OMS had significant antimicrobial effects against Mabc infection in both immunocompetent and immunodeficient mice, and in macrophages. OMS treatment amplified Mabc-induced expression of M1-related proinflammatory cytokines and inducible nitric oxide synthase, and significantly downregulated arginase-1 expression in murine macrophages. In addition, OMS augmented Mabc-mediated production of mitochondrial reactive oxygen species (mtROS), which promoted M1-like proinflammatory responses in Mabc-infected macrophages. OMS-induced production of mtROS and nitric oxide was critical for OMS-mediated antimicrobial responses during Mabc infections. Notably, the combination of OMS and rifabutin had a synergistic effect on the antimicrobial responses against Mabc infections *in vitro*, in murine macrophages, and in zebrafish models *in vivo*. Collectively, these data strongly suggest that OMS may be an effective M1-like adjunctive therapeutic against Mabc infections, either alone or in combination with antibiotics.

## Introduction

Non-tuberculous mycobacteria (NTMs) are emerging as crucial pathogens that cause pulmonary and extrapulmonary diseases worldwide [1–4]. NTM are ubiquitous in the environment and were thought to be opportunistic in immunocompromised patients. However, recent findings suggest that they may cause diseases in immunocompetent individuals [2] and may also be transmissible [5]. The incidence and mortality rates of NTM diseases are increasing, which is imposing a major burden on healthcare systems in developed countries [6]. NTMs are classified as rapidly growing mycobacteria (≤7 days) and slow-growing mycobacteria (>7 days) [7]. Rapidly growing mycobacteria include the *Mycobacteroides abscessus* complex (MAC), a clinically important species accounting for 13% of all NTM pulmonary diseases that has a higher incidence in Asia [7–10]. There are three MAC subspecies, *Mycobacteroides abscessus* (Mabc), *M. massiliense*, and *M. bolletii* [11], according to new genus nomenclature for these strains based on specific molecular markers [6]. Treatment of Mabc-induced pulmonary disease is often challenging because of high rates of antibiotic resistance, frequent relapses, and low cure rates [10,12]. There is an urgent need for novel therapeutic strategies to overcome the current limitations in the treatment of Mabc pulmonary infections.

Macrophages in the innate immune system are responsible for primary defence against a variety of pathogens, including NTMs [13]. They recognize pathogen-associated molecular patterns via innate immune receptors, and trigger complicated molecular signaling pathways to induce antimicrobial and proinflammatory responses against NTM infections [13–15]. However, NTM bacteria can manipulate host innate defense mechanisms in various ways to escape them and establish chronic infections. Notably, the administration of anti-tumor necrosis factor (TNF) or other immunosuppressive drugs is associated with increased susceptibility to NTM diseases [16,17]. In addition, anti-interferon (IFN)-γ autoantibodies are found in NTM pulmonary disease patients [18]. IFN-γ, produced by natural killer cells, promotes M1-mediated innate immune responses against NTM and the production of interleukin (IL)-12 in macrophages [19]. Recent studies have demonstrated that deficiency of a non-histone nuclear protein, HMGN2 (high-mobility group N2), enhances antimicrobial responses to NTM infections through M1 macrophage polarization [20]. These data collectively indicate that M1 macrophage-induced proinflammatory pathways are critical for innate immune responses in the antimicrobial host defense against Mabc infections.

We previously reported that Ohmyungsamycin A (OMS), a cytotoxic and antimicrobial cyclic peptide derived from a marine bacteria belonging to the genus *Streptomyces* [21], exhibited antimicrobial activity against *Mycobacterium tuberculosis* in macrophages through activation of autophagy [22]. In a previous paper on the binding analysis for OMS-A and ecumicin, the macrocyclic depsipeptide, OMS-A can bind to ClpC1 of *M. tuberculosis*, although the binding affinity is substantially lower than ecumicin [23]. A recent study also found that OMS has *in vitro* anti-mycobacterial activity against NTM species, including *M. avium*, *M. intracellulare*, and Mabc [24]. In addition, OMS stimulates *in vitro* and *in vivo* anticancer activities against colorectal cancer cells [25]. However, it remains unclear whether OMS has antimicrobial and immunomodulatory properties in mammalian cells and mouse Mabc pulmonary disease model. Therefore, we first determined the dose of OMS that did not cause cytotoxicity upon macrophage infection with Mabc. We then found that OMS treatment at this safe dose (5 μM) significantly suppressed intracellular Mabc growth in murine bone marrow-derived macrophages (BMDMs), and the lungs of both immunocompetent and immunodeficient mouse models with Mabc pulmonary infections. We further demonstrated that OMS treatment increased the expression of M1-related proinflammatory cytokines, inducible nitric oxide synthase (iNOS), and mitochondrial reactive oxygen species (mtROS) in BMDMs. OMS-mediated mtROS was required to upregulate proinflammatory cytokine expression in macrophages during Mabc infection. Moreover, OMS-induced mtROS and nitric oxide (NO) contributed to Mabc-induced antimicrobial responses. Finally, the combination of OMS and rifabutin (RFB) led to synergistic antimicrobial responses against Mabc infections, *in vitro* and zebrafish (ZF) models. These data suggest that OMS is a potential M1-like adjunctive therapeutic for Mabc infections, either alone or in combination with other antibiotics.

## Materials and methods

### Mice

In this study, sex-matched mice with the age of 6–9 weeks were used. C57BL/6 and BALB/c mice were obtained from the Samtako Bio Korea. C3HeB/FeJ mice were obtained from the Jackson Laboratory. All animal-related procedures were reviewed and approved by the Institutional Animal Care and Use Committee at Chungnam National University (202109A-CNU-180; South Korea). All mice experiments were conducted following the guidelines of the Korean Food and Drug Administration.

### OMS isolation

The bacteria strain stocks of *Streptomyces* sp. SNJ042 were cultured in 50 mL of A1C medium (10 g of starch, 4 g of yeast, 2 g of peptone, 1 g of CaCO_3_, and 27 g of artificial sea salt per 1 L) in a 125-mL Erlenmeyer flask. After 2 days of incubation at 30°C, while shaking at 200 rpm, 10 mL of the liquid culture was inoculated into 200 mL of A1C medium in 500-mL flasks. After 2 days of incubation at 30°C and 170 rpm, 15 mL of the liquid culture was transferred to 1.5 L of A1C medium supplemented with five amino acids (leucine, threonine, valine, phenylalanine, and tryptophan; 2 g/L each) in 2.8-L Fernbach flasks for optimizing the production of OMS [26]. The culture was maintained for 6 days before a separation funnel was used to extract the entire 200 L with 300 L of ethyl acetate. After removing water by adding anhydrous sodium sulfate, the liquid extract was concentrated in *vacuo* to yield 30 g of dry extract.

The crude extract was fractionated through a C-18 reversed-phase open column using 20%, 40%, 60%, 80%, and 100% aqueous MeOH. The 80% MeOH fraction containing OMS was dried and injected directly into a preparative reversed-phase high-performance liquid chromatography (HPLC) column (Phenomenex C_18_(2) Luna, 10 μm, 250 × 21.2 mm; flow rate: 10 mL/min, detection: UV = 280 nm) using a gradient of 40–55% aqueous CH_3_CN (0.1% trifluoroacetic acid) for 20 min, followed by isocratic 60% aqueous CH_3_CN (0.1% trifluoroacetic acid). OMS-A was eluted at 24 min and further purified using a reversed-phase HPLC column (Kromasil C_18_, 5 μm, 250 × 10 mm) with a gradient of 40–60% aqueous CH_3_CN (0.1% trifluoroacetic acid) for 30 min, followed by isocratic 60% aqueous CH_3_CN (0.1% trifluoroacetic acid). Pure OMS-A was obtained at 36 min. This procedure was repeated to collect 400 mg of OMS-A.

### Mycobacterial strain and culture

The smooth ATCC19977 strain of Mabc, and Mabc *subsp. abscessus* CIP 104536T S and R morphotypes tagged with mWasabi were used for this study. Mycobacteria were grown at 37°C with shaking in Middlebrook 7H9 broth (271310; BD Difco) containing 0.2% glycerol and 10% oleic albumin dextrose catalase (OADC; BD) until the mid-logarithmic phase (OD_600 nm_ = 0.4). After cultivation, bacteria culture media were harvested by centrifugation at 3000 rpm for 20 min and washed three times with cold PBS to completely remove the BSA and glycerol. Mycobacterial suspensions were aliquoted and stored at −80°C.

### Cell culture

Bone marrow-derived macrophages (BMDMs) were harvested from the C57BL/6 wild-type mice (6–8 weeks of age) and cultured as previously described [27]. Briefly, BMDMs were prepared in DMEM supplemented with 10% FBS (16000–044; Gibco), antibiotics (17-745E; Lonza), and 25 ng/mL macrophage colony-stimulating factor recombinant mouse (416-ML; R&D Systems) for 6–7 days in a 37°C incubator. To harvest peritoneal macrophages, 1 mL of 3% of thioglycolate solution was intraperitoneally injected into mice. Three days after the injection, peritoneal fluids were harvested in cold PBS containing 2% FBS. The collected fluids were centrifuged and the cells were cultured overnight in DMEM for further experiments [28].

### *Mouse* in vivo *infection model*

C57BL/6 and C3HeB/FeJ mice (6–8 weeks of age) were anesthetized and challenged with Mabc (1 × 10^7^ colony forming units (CFU)/mouse for C57BL/6 and 1 × 10^4^ CFU/mouse for C3HeB/FeJ) intranasally. BALB/c mice were pre-treated daily with or without dexamethasone (5 mg/kg, subcutaneously). After 2 weeks, the mice were anesthetized and challenged with Mabc (1 × 10^3^ CFU/mouse) intranasally. The lung tissues were collected on days 14 and 21 after infection as indicated in the figures, and homogenized with an Omni Tissue homogenizer (PerkinElmer) in PBS. The homogenates were serially diluted in PBST and plated on 7H10 agar plates. Mabc colonies were counted to measure the bacterial loads after 3–4 days of incubation.

### Intracellular CFU assay

BMDMs (1 × 10^5^ cells) plated in a 48-well plate were infected with Mabc (MOI of 1) for 2 h at 37°C. After the infections, extracellular bacteria were washed away with warmed PBS, and the infected cells were further cultured for 24 h or 48 h in DMEM containing 5% FBS. BAY 11–7082 (196870, Calbiochem), N^G^-Methyl-L-arginine acetate salt (L-NMMA) (M7033, Sigma), TPCA-1 (401481, Calbiochem), MitoTEMPO (SML0737, Sigma) were store at −20°C and sonicated right before each use. To count the intracellular bacteria, BMDMs were lysed by distilled water, followed by serial dilution in PBS, and then plated in 7H10 agar plates and incubated at 37°C. Mabc colonies were counted to determine the bacterial loads after 3–4 days of incubation.

### Histology and immunochemistry

C57BL/6 mice lung tissues were collected after 21 days of Mabc infection. Lung tissues were fixed in 10% formalin, embedded in paraffin wax, and cut into 4 μm sections. The lung sections (4 μm) were stained with hematoxylin and eosin (H&E). Slides were digitalized by scanner PANNORAMIC 300 Flash DX device (3DHISTECH Ltd), and the inflamed area was quantified by using Fiji software. For IHC staining, lung paraffin sections (4 μm) were stained with 4′,6-diamidino-2-phenylindole DAPI (P36935; Invitrogen), anti-NOS2 mouse (sc-7271; Santa Cruz Biotechnology), anti-F4/80 rat (sc-52664; Santa Cruz Biotechnology), and matched secondary antibodies. (A11029 and A21209; Invitrogen) Immunochemical staining images were obtained by a Leica TCS SP8 microscope system (Leica) [29].

### Cell viability

Cell viability was determined by Cell Counting Kit-8 (CCK-8) (CK04–01; Dojindo). BMDMs (5 × 10^4^ cells) were cultured in a 96-well flat-bottomed plate at 37°C. After infection with Mabc in the presence or absence of OMS for 24 h, CCK-8 dye was added and incubated at 37°C for 2 h. The absorbance was assessed at 450 nm using a microplate reader.

### RNA extraction and real-time quantitative PCR

Total RNA from BMDMs or PMs or lung tissues were isolated using TRIzol reagent (15596026; Invitrogen). Complementary DNA (cDNA) was synthesized by Reverse Transcriptase Premix (EBT-1515; Elpis Biotech). Quantitative PCR (qPCR) was carried out using QuantiNova PCR kits (208056; Qiagen) as recommended by the manufacturer. qPCR data were analyzed by the 2^–ΔΔCt^ (Ct) method with mouse *Actin* as an internal control gene. Primers sequences (mouse) used in this study were as follows: *Tnf* forward: 5’-ACGGCATGGATCTCAAAGAC-3', reverse: 5’-AGATAGCAAATCGGCTGACG-3'; *Il1b* forward: 5’-TACGGACCCCAAAAGATGA-3', reverse: 5’- TGCTGCTGCGAGATTTGAAG-3'; *Il12p40* forward: 5’- TTGAACTGGCGTTGGAAGCACG-3', reverse: 5’- CCACCTGTGAGTTCTTCAAAGGC-3'; *Ccl5* forward: 5’- CCTGCTGCTTTGCCTACCTCTC-3', reverse: 5’- ACACACTTGGCGGTTCCTTCGA-3'; *Nos2* forward: 5’- GGTGAAGGGACTGAGCTGTTA-3,' reverse: 5’- TGAAGAGAAACTTCCAGGGGC-3'; *Arg1* forward: 5’- CTCCAAGCCAAAGTCCTTAGAG-3', reverse: 5’- AGGAGCTGTCATTAGGGACATC-3'; *Il10* forward: 5’- GCTCTTGCACTACCAAAGCC-3', reverse: 5’- CTGCTGATCCTCATGCCAGT-3'; *Actin* forward: 5’- CCACCATGTACCCAGGCATT-3', reverse: 5’- AGGGTGTAAAACGCAGCTCA-3'.

### Western blot analysis

For Western blot analysis, proteins samples were subjected to SDS-PAGE and then transferred on PVDF membrane (Millipore). Membranes were blocked in 5% skim milk in TBS-T for 1 h at room temperature. After blocking, Membranes were incubated overnight with primary antibodies aginst at phospho-NF-κB p65 (3033, Cell Signaling), phospho-ERK1/2 (9101, Cell Signaling), phospho-SAPK/JNK (4668, Cell Signaling), phospho-p38-MAPK (9211, Cell Signaling) and Actin (sc-47778, Santa Cruz Biotechnology) at 4°C, followed by second incubation at room temperature for 1 h with appropriate HRP-conjugated secondary antibodies (Cell Signaling). The immune-reactive proteins were detected using chemiluminescence kit. ImageJ software was used for densitometry analysis.

### Enzyme-linked Immunosorbent Assay (ELISA)

PMs (1.5 × 10^5^ cells) were cultured in 48-well plate and infected with Mabc (MOI of 1) with or without OMS treatment (5 μM). Cell supernatants were then collected after 3–18 h to measure TNF using the Mouse TNF ELISA kit (558534; BD Biosciences) according to the manufacturer’s protocol.

### Evaluation of drug effects using REMA chequerboard assay

Resazurin microtiter assay (REMA) and chequerboard assay were performed as described previously [30,31]. The minimum inhibitory concentration 50 (MIC_50_) was defined as the minimum concentration required to inhibit 50% growth of the organism. To assess the interactions between compounds, following formula was used to calculate fractional inhibitory concentrations (FICs): FIC (X + Y) = [MIC of compound X in combination with Y]/[MIC of X alone]. The fractional inhibitory index (ΣFIC) is the sum of the FIC of compounds X + FIC of compound Y. ΣFIC value ≤0.5 designate synergistic activity [32]. Bacteria incubated in the presence of combination of drugs were inoculated on 7H10 agar plates. Mabc colonies were counted after 3–4 days of incubation, and the bacterial viability was calculated.

### Flow cytometry

To analyze M1-like macrophages, BMDMs were stained with PerCP/Cyanine5.5 anti-mouse/human CD11b (101228; BioLegend), APC anti-mouse F4/80 (123116; BioLegend), and Alexa Fluor 488® anti-mouse CD86 (105017; BioLegend). For intracellular iNOS staining, BMDMs after Fc-blocking were stained with PerCP/Cyanine5.5 anti-mouse/human CD11b (101228; BioLegend) and APC anti-mouse F4/80 (123116; BioLegend) at 4°C for 30 min, fixed and permeabilized with BD Cytofix/Cytoperm Kit (554714; BD Biosciences) at 4°C for 20 min. After washing with Perm/Wash buffer (BD Biosciences), the cells were stained with Alexa Fluor 488 anti-iNOS (53-5920-80; Invitrogen) at 4°C for 30 min. The stained cells were resuspended in 2% paraformaldehyde and analyzed using the NovoCyte 2060 R (ACEA Biosciences, USA), using FlowJo software ver 10.7.2 (BD Biosciences)

### mtROS and NO production

To quantify mtROS, BMDMs were loaded with 2.5 μM MitoSOX^TM^ solution (M36008; Invitrogen) at 37°C for 20 min. After incubation, cells were measured using confocal microscope ZEISS LSM 900. MitoSOX intensity analyzed with MetaMorph software. BMDMs were infected Mabc (MOI of 1) to measure NO production and then incubated with or without OMS (5 μM) for 72 and 96 h. Cell supernatant was harvested and the nitrite concentration was measured by the Griess Reagent Kit (G7921; Invitrogen), as described in the manufacturer’s protocol.

### Zebrafish infection and drug treatment

The Animal Research Ethics Committee at Gyeongsang National University (GNU-190325-E0014, South Korea) authorized all ZF studies and research procedures. ZF infection was performed as described previously [31]. mWasabi-labeled Mabc CIP 104536T R morphotype was used for the fluorescence study. The infected larvae were transferred into 96 well plates, with two fish per well and exposed to the drug combination. The drug combination solution and water were renewed daily. For the negative control, DMSO-treated ZF larvae were used.

### Drug eﬃcacy assessment in Mabc-infected ZF

*In vivo* drug combination efficacy was determined based on GFP-labeled Mabc proliferation in ZF, CFU counting after homogenization of infected/treated ZF, and the survival curve. Bacterial proliferation was monitored by analyzing Mabc-GFP evolution inside the infected ZF larvae at 5 dpi using the ImageXpress Pico Automated Cell Imaging System (Molecular Devices). For bacterial burden quantification after treatment, groups of 20 infected embryos were collected 5 days after infection, lysed individually in 2% TritonX-100 PBST and were homogenized with a handheld homogenizer (D1000; Benchmark). Ten-fold serial dilutions of homogenates were plated on Middlebrook 7H10 Agar (262710; BD Biosciences) containing 50 µg/mL kanamycin and a Mycobacteria Growth Indicator BBL™ MGIT™ PANTA (BD Biosciences). After 3–5 days of incubation at 37ºC, colonies were counted. The Kaplan–Meier survival curve was determined by recording dead embryos daily for 13 days.

### Statistical analyses

For statistical analysis, Prism (GraphPad version 8.4.0.) was used. Data obtained from independent experiments [mean ± standard deviation (SD) or standard error of the mean (SEM)] were analysed using a two-tailed Student’s *t*-test and one-way analysis of variance (ANOVA) with Tukey’s multiple comparison test. Specific *p* values are detailed in the figure legends. *p < 0.05, **p < 0.01, and ***p < 0.001 were considered indicative of statistical significance. Kaplan–Meier survival curves were generated and analyzed by a Gehan-Breslow-Wilcoxon test for the ZF survival test.

## Results

### *OMS promoted* in vitro *and* in vivo *antimicrobial responses in Mabc infections*

**Figure 1.**
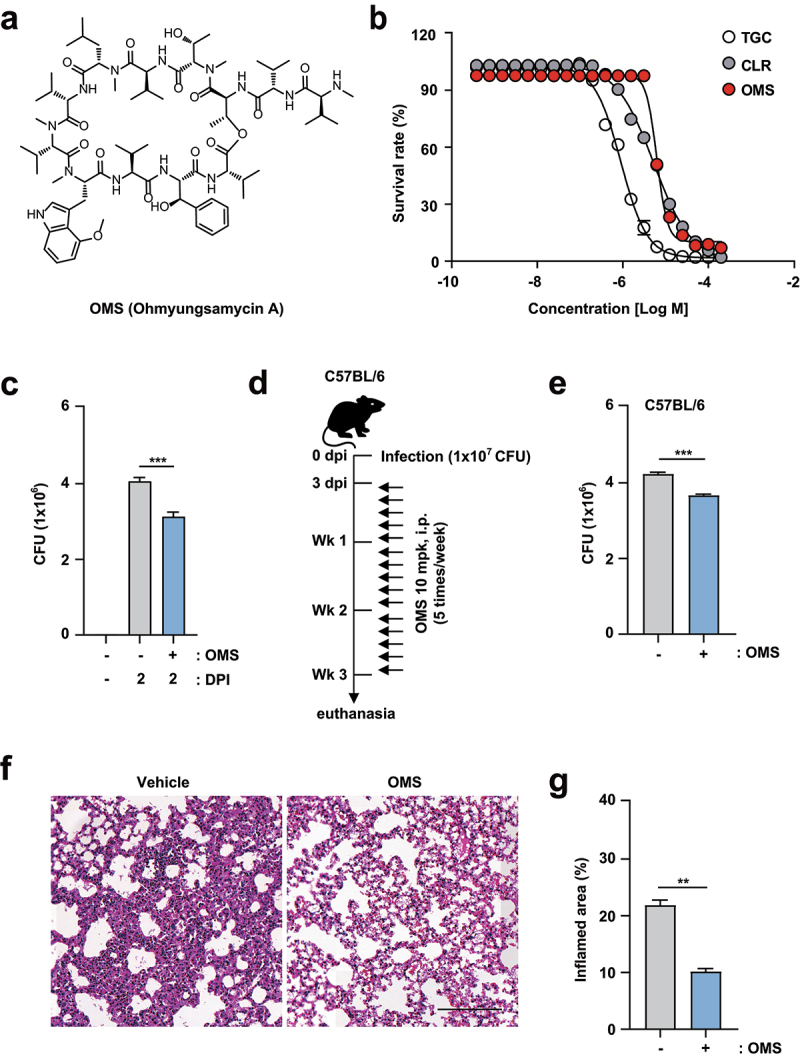
OMS treatment boosts antimicrobial responses against Mabc infections *in vitro* and *in vivo*. (a) Structure of OMS-A. (b) REMA comparing the sensitivity of OMS with CLR and TGC. (c) Intracellular survival assay for Mabc (MOI of 1) in BMDMs with or without OMS treatment (5 μM) for 2 days. (d and e) C57BL/6 mice were infected intranasally with Mabc (1 × 10^7^ CFU), treated with or without OMS (five times a week; 10 mg/kg i.p.) and monitored at 21 dpi. Schematic diagram of the experimental design (c) and CFU assay of lung tissues (e). (f and g) Representative H&E stained images (f, scale bar = 300 μm) of the lung tissue of mice treated as in (d), and quantitative analysis of the inflamed area (f). Statistical significance was assessed using the two-tailed Student’s *t*-test (b, d, f). Data are representative of at least three independent experiments, and are presented as means ± SD or SEM. ** *p* <0.01, *** *p* <0.001. dpi, days post infection; CFU, colony forming units; OMS, ohmyungsamycin A, CLR, clarithromycin; TGC, tigecycline.

Previous studies have reported antimicrobial activity of OMS against *M. tuberculosis* (Mtb) and NTMs, including Mabc [22,24]. Therefore, we first confirmed the MIC of the newly purified OMS-A against Mabc using REMA [26]. Structure of OMS used in this study is shown in Figure 1(a). Clarithromycin and tigecycline were selected as positive controls. After incubating Mabc for 3 days with the compounds, a sigmoidal dose response curve was plotted, and indicated a concentration-dependent inhibitory effect. Mabc was susceptible to OMS and the MIC_50_ of OMS for Mabc was 6.4 μM (Figure 1(b)).

As the antimicrobial effects of OMS in mammalian cells were unclear, we determined the safe OMS concentrations in mammalian cells using a CCK8 assay. Overall, OMS exhibited no toxicity up to 5 µM in BMDMs, regardless of Mabc infection. However, OMS concentrations above 10 µM were strongly cytotoxic in Mabc-infected BMDMs (MOI of 1; Supplementary Figure S1). An OMS concentration of 5 µM was used for the study, because it had optimal in vitro antimicrobial effects without significant cellular cytotoxicity. We further determined the OMS effects on intracellular Mabc survival by intracellular CFU assay of BMDMs. OMS treatment significantly inhibited intracellular Mabc survival at 2 dpi in BMDMs (Figure 1(c)).

To assess the *in vivo* effectiveness of OMS, C57BL/6 mice were infected with Mabc via intranasal inoculation at a dose of 1 × 10^7^ CFU in 25 μL of saline, and treated with OMS (10 mg/kg i.p.) for 5 consecutive days. The *in vivo* experimental conditions for C57BL/6 mice are shown in Figure 1(d). At 21 dpi, Mabc growth was significantly reduced by OMS treatment in the lung tissues from C57BL/6 mice (Figure 1(e)). Histopathological changes in lung sections from C57BL/6 mice were further examined by H&E staining. At 21 dpi, the lungs of OMS-treated mice showed fewer granulomatous lesions compared to PBS-treated controls (Figure 1(f,g)).

**Figure 2.**
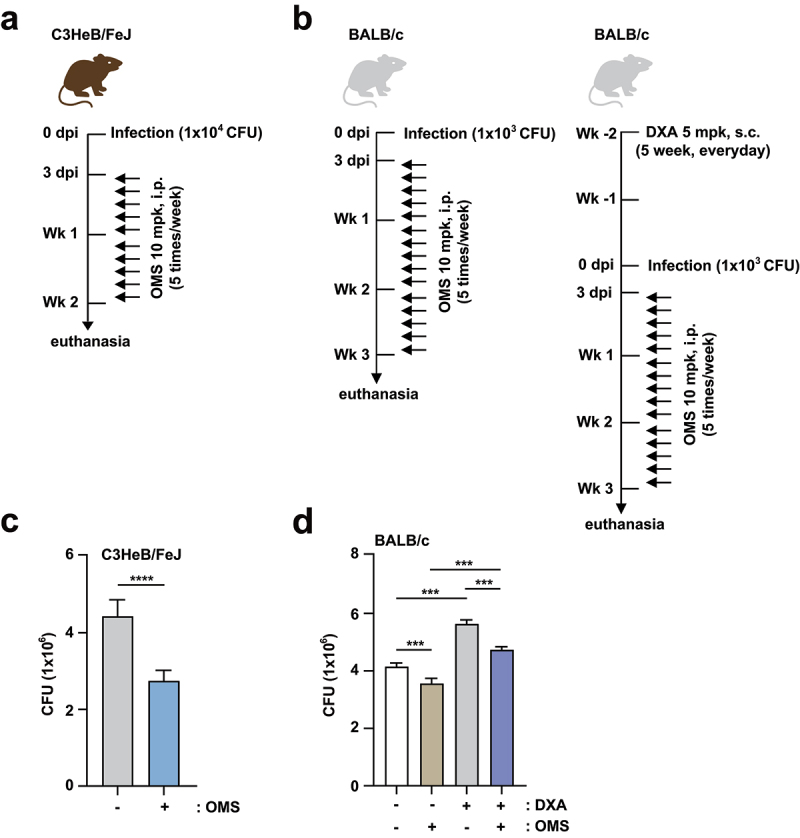
OMS treatment promotes antimicrobial responses against Mabc infection in immunocompetent and immunodeficient mouse models.(a, c) C3HeB/FeJ mice were infected intranasally with Mabc (1 × 10^4^ CFU), with or without OMS treatment (five times a week; 10 mg/kg i.P.), and monitored at 14 dpi. Schematic diagram of the immunocompetent mouse model design (a) and CFU assay of lung tissues (c). (b, d) BALB/C mice were treated with or without dexamethasone (daily s.c. 5 mg/kg) over 2 weeks. After treatment, the mice were infected intranasally with Mabc (1 × 10^3^ CFU), treated with or without OMS (five times a week; 10 mg/kg i.P.), and monitored at 21 dpi. Schematic diagram of the immunodeficient mouse model (b) and CFU assay in the lung tissues (d). Statistical significance was assessed using the two-tailed Student’s *t*-test (c) and one-way ANOVA with Tukey’s multiple comparison test (d). Data are means ± SEM of three independent experiments. *** *p* < 0.001. dpi, days post infection; CFU, colony forming units; OMS, ohmyungsamycin A; DXA, dexamethasone.

Furthermore, we examined whether OMS treatment increased antimicrobial responses in either C3HeB/FeJ or BALB/c mice. The *in vivo* experimental conditions for C3HeB/FeJ and BALB/c mice are shown in Figure 2(a,b), respectively. For Mabc infection, BALB/c mice were divided into two groups (with and without dexamethasone pretreatment) to examine the effects of OMS in both immunocompetent and immunocompromised mice (Figure 2(b)). CFU assay showed that OMS treatment significantly reduced the bacterial loads of Mabc in the lungs from C3HeB/FeJ mice (Figure 2(c)), which are susceptible to Mabc infections [33]. Mabc growth was also suppressed by OMS administration in BALB/c mice, and this attenuation was most profound with dexamethasone pretreatment (Figure 2(d)). Together, these results indicated significant reductions in the Mabc burden and pathological lesions in the lungs of OMS-treated mice.

### *OMS treatment increased M1-related proinflammatory responses* in vivo, *and in Mabc-infected macrophages*

**Figure 3.**
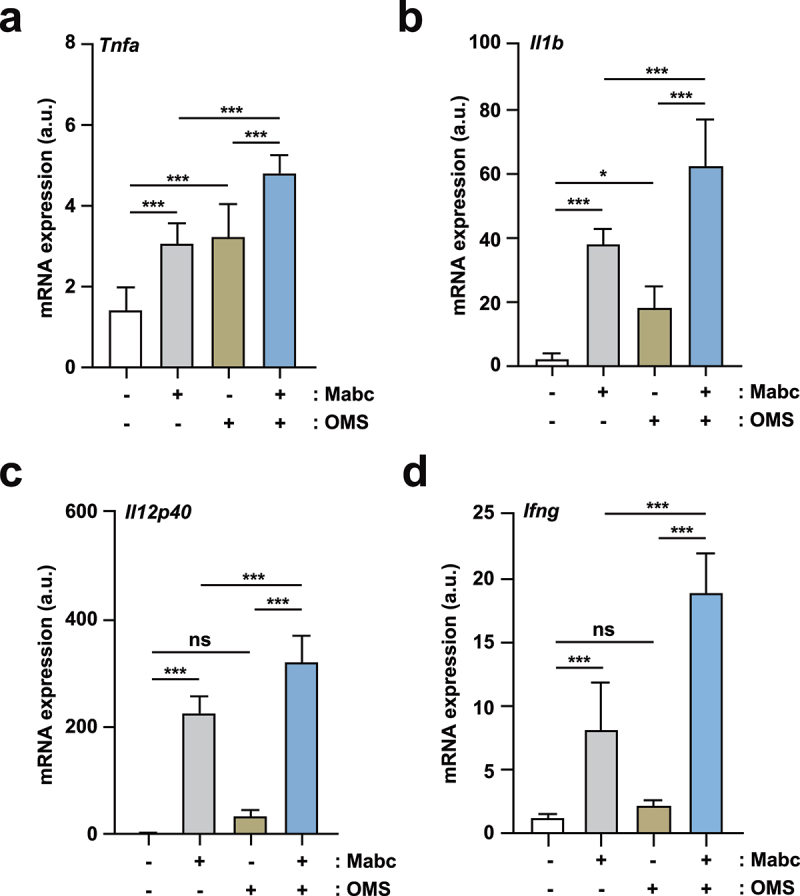
OMS treatment induces M1-related proinflammatory responses in Mabc-infected mice *in vivo*.(a – d) qPCR analysis of *Tnf*, *Il1b*, *Il12p40*, and *Ifng* mRNA expression in C57BL/6 mice lung tissues. C57BL/6 mice were infected intranasally with Mabc (1 × 10^7^ CFU), with or without OMS treatment (five times a week; 10 mg/kg i.p.), and monitored at 7 dpi (a – c) and 21 dpi (d). Statistical significance was assessed using one-way ANOVA with Tukey’s multiple comparison test (a – d). Data are means ± SEM of three independent experiments. * *p* < 0.05, *** *p* < 0.001. dpi, days post infection; OMS, ohmyungsamycin A; a.u., arbitrary unit; n.s., not significant. .

To determine the effects of OMS on Mabc-induced inflammation, proinflammatory cytokine expression was assessed by measuring the production of key cytokine mRNAs and proteins in infected lungs and macrophages. We first examined the mRNA expression levels of M1-related proinflammatory cytokines/chemokines (*Tnf*, *Il1b*, and *Il12p40*) in lung tissues from Mabc-infected mice treated with or without OMS at 7 dpi. mRNA expression levels of M1-associated *Tnf*, *Il1b*, and *Il12p40* were significantly increased in the lungs of Mabc-infected mice (Figure 3(a,b,c)). In addition, OMS treatment significantly augmented proinflammatory cytokine levels in lung tissues from Mabc-infected mice (Figure 3(a,b,c)). Moreover, *Ifng* mRNA levels were measured in lung tissues from Mabc-infected mice treated with or without OMS at 21 dpi. OMS treatment markedly upregulated *Ifng* mRNA expression in the lungs of Mabc-infected mice (Figure 3(d)), suggesting that OMS treatment upregulates Th1 responses in late phases of *in vivo* Mabc infection.

**Figure 4.**
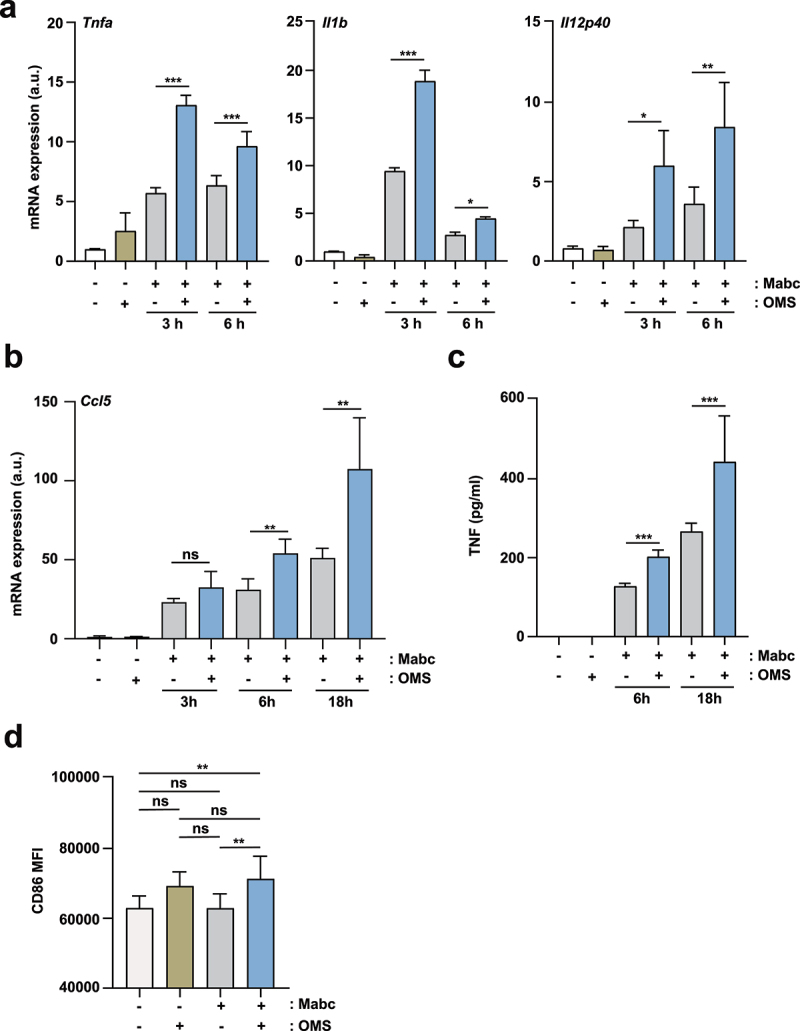
OMS treatment induces an M1-like state in macrophages during Mabc infection.(a, b) qPCR analysis of *Tnf, Il1b, Il12p40* (a), and *Ccl5* (b) mRNA expression in BMDMs. BMDMs were infected with Mabc (MOI of 3), with or without OMS treatment (5 μM), for the indicated times. (c) ELISA of TNF in the supernatant from PMs. PMs were infected with Mabc (MOI of 1), with or without OMS treatment (5 μM) for the indicated times. (d, e) Flow cytometry of CD86 expression in BMDMs. BMDMs were infected with Mabc (MOI of 1), with or without OMS treatment (5 μM) for 3 days. (d) Mean fluorescence intensity of CD86. Statistical significance was assessed using one-way ANOVA with Tukey’s multiple comparison test (a – c, d). Data are means ± SD of three independent experiments. * p < 0.05, ** p < 0.01, *** p < 0.001. BMDMs, bone marrow derived macrophages, a.u., arbitrary unit.

We then examined whether proinflammatory cytokine production was increased by OMS treatment in Mabc-infected BMDMs or PMs. At 3 and 6 h post-infection, OMS-treated BMDMs and PMs had significantly higher mRNA levels of *Tnf*, *Il1b*, and *Il12p40* compared to untreated controls (Figure 4(a) and Supplementary Figure S2). OMS treatment also significantly increased the mRNA expression of M1-driving chemokine C-C motif chemokine ligand 5 (CCL5) in Mabc-infected BMDMs at 6 and 18 h compared to untreated controls (Figure 4(b)). In addition, OMS treatment induced greater TNF-α production in Mabc-infected PMs at 6 and 18 h compared to untreated PMs (Figure 4(c)). Flow cytometry showed that OMS treatment significantly augmented CD86 expression in Mabc-infected BMDMs compared to untreated BMDMs (Figure 4(d)). Collectively, these data suggest that OMS treatment markedly amplifies M1-related proinflammatory responses *in vivo*, and in macrophages during Mabc infection.

### Macrophage nuclear factor-κB pathway activation contributes to OMS-induced antimicrobial responses against Mabc infection

**Figure 5.**
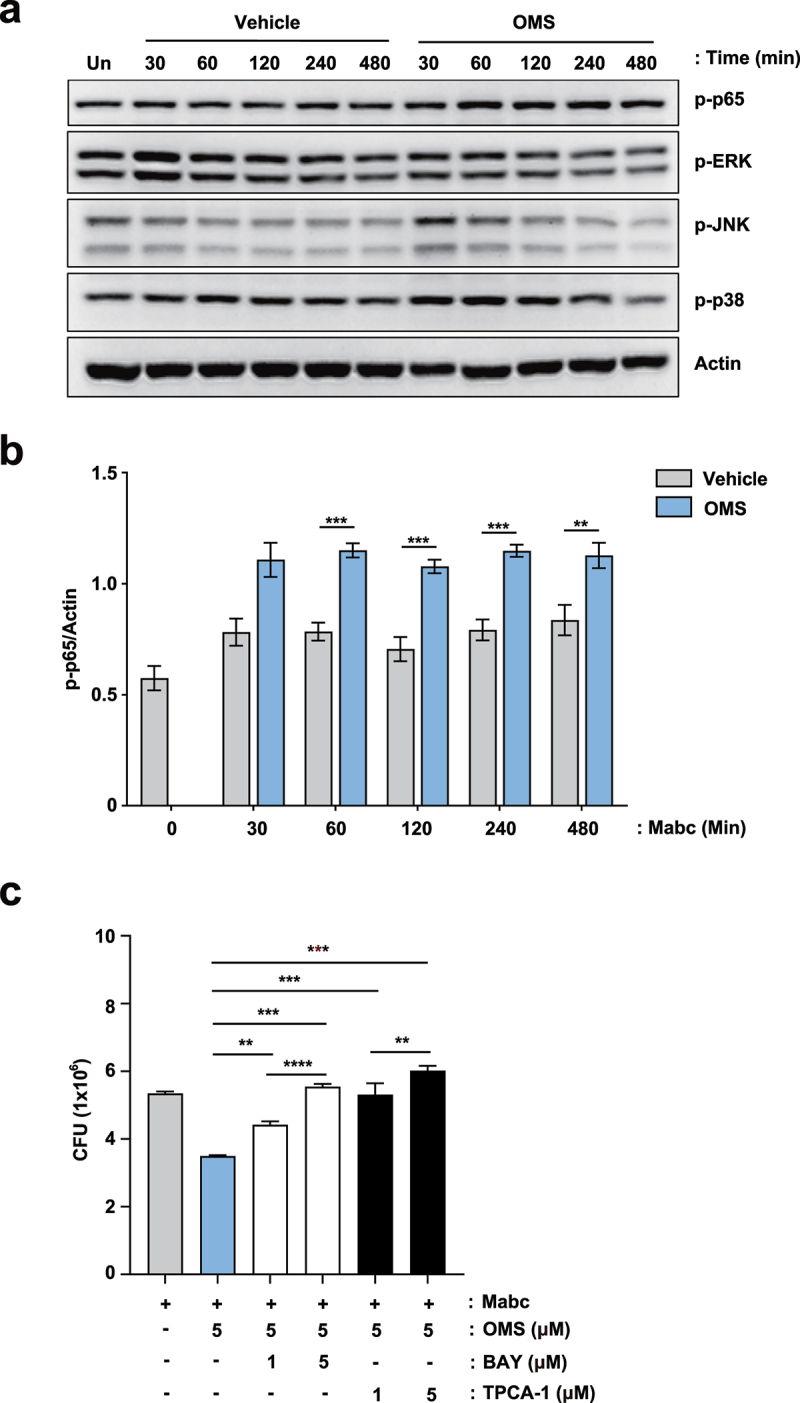
NF-κB signalling pathway activation contributes to OMS-induced antimicrobial responses in BMDMs during Mabc infection. (a) Representative immunoblots in BMDMs. BMDMs were infected with Mabc (MOI of 1), with or without OMS treatment (5 μM) for the indicated times and probed with antibody to phospho (p)-p65, p-ERK1/2 (p-ERK), p-JNK1/2 (p-JNK), p-p38 MAPK (p-p38) or actin. (b) Densitometric quantifications of p-p65. (c) Intracellular survival assay in BMDMs. BMDMs were pre-incubated for 1 h with or without indicated concentration (1 or 5 μM) of either BAY or TPCA-1, followed by infection with Mabc (MOI of 1). Cells were then treated with OMS (5 μM) in the presence or absence of either BAY or TPCA-1 (1 or 5 μM) for 2 days. Statistical significance was calculated using unpaired student *t*-test (for b) and One-way ANOVA with Tukey’s multiple comparison test (for c). Data are presented as mean ±SD. OMS, Ohmyungsamycin A; BAY, BAY 11–7082; CFU, colony forming units; n.s., not significant; *, P < 0.05; **, P < 0.01; ***, P < 0.001.

To examine whether the inflammatory signaling pathway is involved in the OMS-induced antimicrobial responses, we first assessed which intracellular signaling pathways are upregulated by OMS treatment in macrophages during infection. As shown in the Figure 5(a,b), the phosphorylation of nuclear factor (NF)-κB p65 subunit was significantly upregulated by OMS treatment in Mabc-infected BMDMs. However, the addition of OMS to Mabc-infected BMDMs resulted in a comparable or reduced level of phosphorylation of mitogen-activated protein kinases [extracellular signal‐regulated kinase 1 and 2 (ERK1/2), p38 MAPK, c‐Jun NH2‐terminal (JNK) MAPK] (Figure 5(a)). We thus selected NF-κB signaling pathway to determine the role for the regulation of antimicrobial responses induced by OMS.

Notably, pre- and post-treatment of inhibitors of NF-κB signaling pathway [BAY 11–7082 (BAY) or TPCA-1] significantly increased the intracellular survival of Mabc in OMS-treated BMDMs. BMDMs were infected with Mabc (MOI of 1) and followed by treatment with OMS in the presence or absence of either BAY or TPCA-1 (well-known inhibitors of NF-κB signaling pathway) for 2 days. The use of pharmacological inhibitors (BAY and TPCA-1) did not affect the cell viability until 48 hr (data not shown). As shown in the previous results, the OMS-treated group showed significantly decreased intracellular survival of Mabc than those from the solvent control group. It was noted that pre- and post-treatment with either BAY or TPCA-1 exhibited significantly reversed OMS-induced antimicrobial responses in BMDMs in a dose-dependent manner (Figure 5(c)). These results suggest that the activation of the NF-κB signaling pathway is at least partly involved in the OMS-induced antimicrobial responses against Mabc infection.

### OMS treatment increased iNOS expression and NO production, but reduced M2 responses during Mabc infection

**Figure 6.**
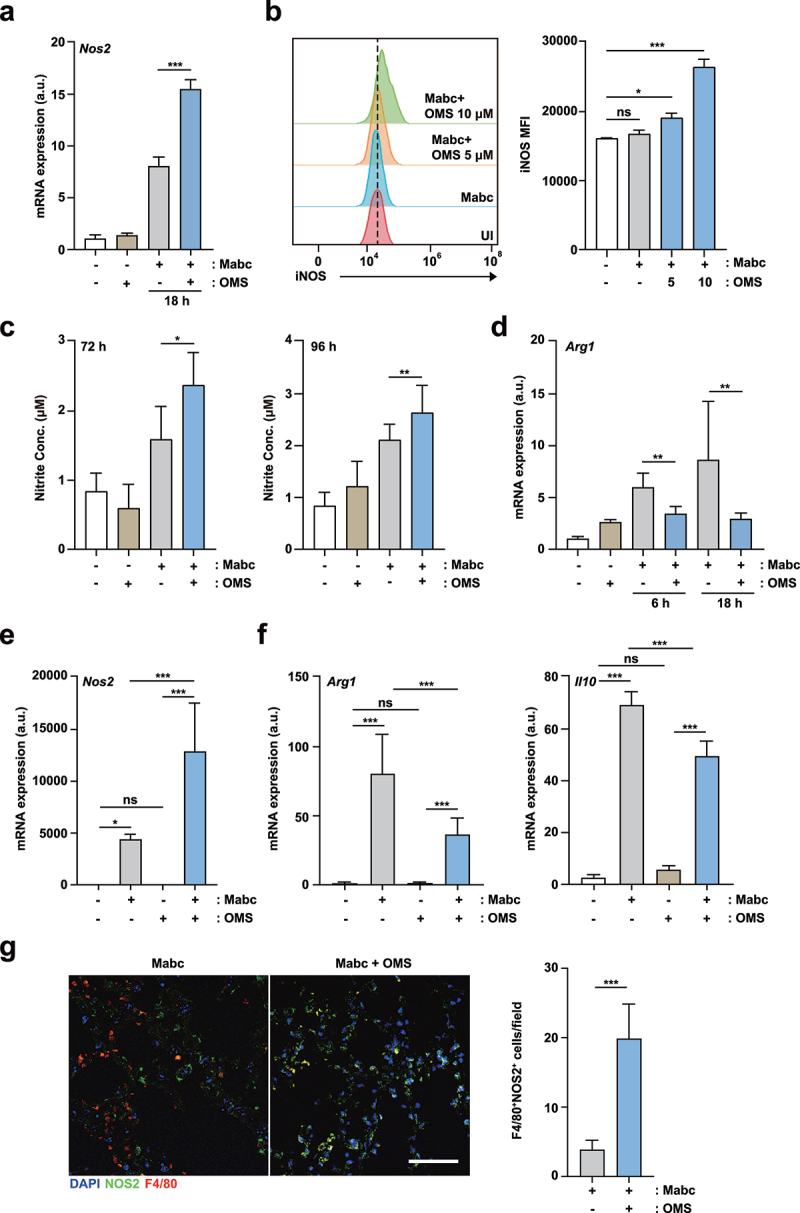
OMS treatment increases iNOS expression and NO production, but reduces M2-like cytokines.(a) qPCR analysis of *Nos2* mRNA expression in BMDMs. BMDMs were infected with Mabc (MOI of 3), with or without OMS treatment (5 μM or 10 μM) for the indicated times. (b) Flow cytometric analysis of iNOS expression in BMDMs. BMDMs were infected with Mabc (MOI of 3), and treated with or without OMS for 2 days. (c) Nitrite was measured in the supernatant from BMDMs using griess assay. BMDMs were infected with Mabc (MOI of 1), with or without OMS treatment (5 μM) for the indicated times. (d) qPCR analysis of *Arg1* mRNA expression in BMDMs, as in (a). (e, f) qPCR analysis of *Nos2*, *Arg1*, and *Il10* mRNA expression in C57BL/6 mouse lung tissues. C57BL/6 mice were infected intranasally with Mabc (1 × 10^7^ CFU), with or without OMS treatment (five times a week; 10 mg/kg i.P.), and monitored at 21 dpi. Statistical significance was assessed using one-way ANOVA with Tukey’s multiple comparison test (a, b right, c – f). (g) Representative immunofluorescence images (scale bar = 50 μm) and quantitative analysis of macrophage iNOS expression (F4/80+ NOS2+ cells) per field of lung tissues from Mabc-infected mice treated with or without OMS. Data are means ± SEM of three independent experiments. * *p* < 0.05, ** *p* < 0.01, *** *p* < 0.001. BMDMs, bone marrow derived macrophages, OMS, Ohmyungsamycin A; a.u., arbitrary unit; n.s., not significant; UI, uninfected.

We assessed the effects of OMS on the expression of the iNOS gene, which is generated by M1 macrophages [34] during Mabc infection. OMS-treated BMDMs showed significantly higher mRNA expression of iNOS during Mabc infection compared to untreated controls (Figure 6(a)). In addition, flow cytometry showed that Mabc-mediated iNOS protein expression was significantly amplified in OMS-treated BMDMs compared to untreated controls (Figure 6(b)). The gating strategies are shown in Supplementary Figure S3.

Free-radical NO expression was measured using Griess reagents [35]. OMS treatment induced significantly higher production of NO in BMDMs at 72 and 96 h after Mabc infection compared to untreated BMDMs (Figure 6(c)). The mRNA expression of arginase-1 (Arg1), which is generally synthesized by alternatively activated M2 macrophages [34], was reduced by OMS treatment in Mabc-infected BMDMs, compared to untreated BMDMs and BMDMs infected with Mabc only (Figure 6(d)). Furthermore, mRNA expression levels of iNOS, Arg1, and IL-10 were compared in the lung tissues of Mabc-infected mice treated with and without OMS at 21 dpi. Mabc-induced *Nos2* expression was significantly increased (Figure 6(e)), whereas the mRNA levels of *Arg1* and *Il10* were significantly downregulated (Figure 6(f)) by OMS treatment. Similarly, macrophage iNOS expression (F4/80+NOS2+ cells) was significantly increased after OMS treatment in the lungs of Mabc-infected mice (Figure 6(g)). These results suggest that OMS treatment promotes iNOS induction, but decreases Arg1 and IL-10 production, in BMDMs during Mabc infection.

### OMS treatment triggered production of the mtROS required for proinflammatory responses during Mabc infection

**Figure 7.**
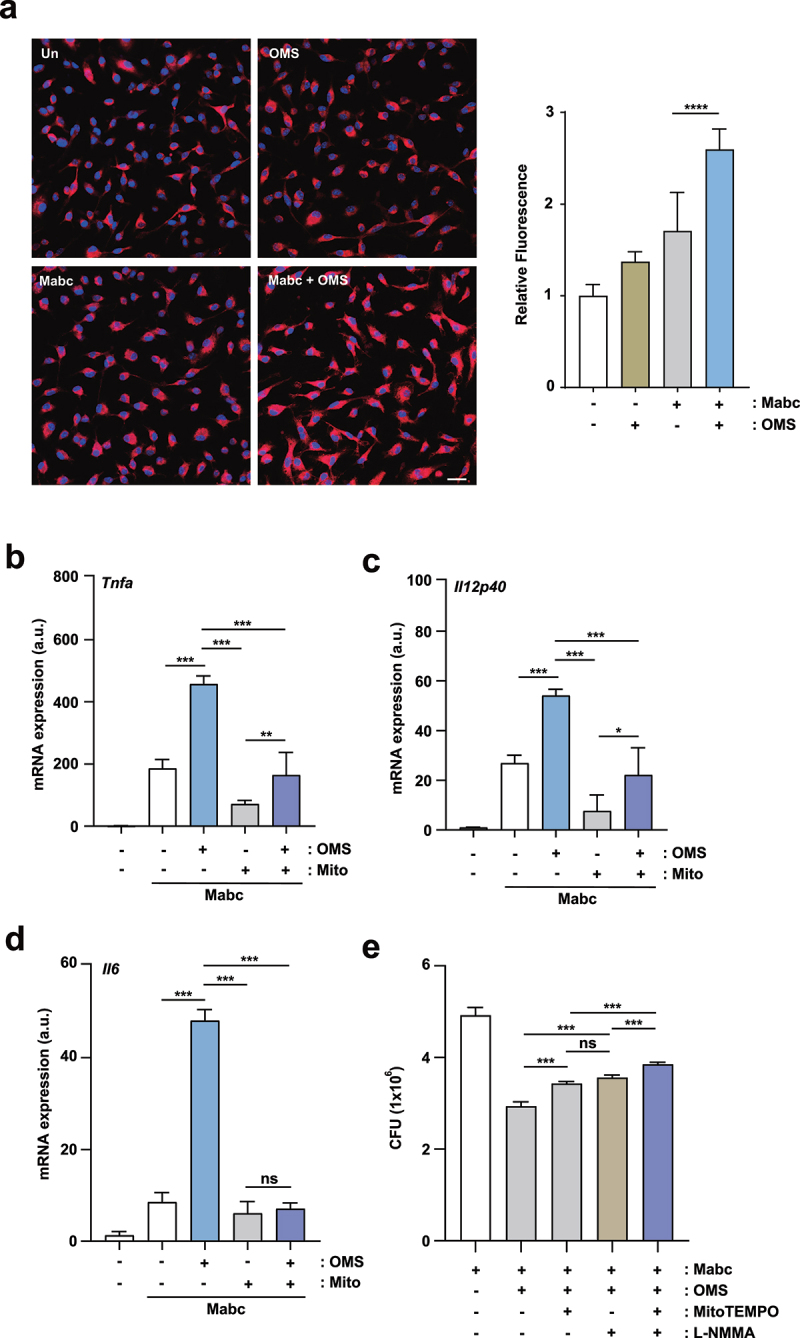
OMS-induced mtROS production mediates inflammatory signalling and antimicrobial responses during Mabc infection. (a) Representative immunofluorescence images (scale bar = 20 μm) and quantitative analysis of relative fluorescence intensities of mtROS. BMDMs were infected with Mabc (MOI of 3), with or without OMS treatment (5 μM) for 2 h. (b – d) qPCR analysis of *Tnf, Il1b*, and *Il6* mRNA expression in BMDMs. BMDMs were infected with Mabc (MOI of 3), and treated with OMS (5 μM) or MitoTEMPO (50 μM) for 6 h. (e) BMDMs were infected with Mabc (MOI of 1), and treated with OMS (5 μM), MitoTEMPO (200 μM), and L-NMMA (1 mM) for 2 days. Intracellular survival was assessed by CFU analysis. Statistical significance was assessed using one-way ANOVA with Tukey’s multiple comparison test (a right, b–e). Data are means ± SD or SEM of three independent experiments. * *p* < 0.05, ** *p* < 0.01, *** *p* < 0.001. **** *p* < 0.0001 BMDMs, bone marrow derived macrophages, OMS, Ohmyungsamycin A; a.u., arbitrary unit; n.s., not significant.

Given that classical macrophage activation promotes antimicrobial defence in an mtROS-dependent manner [36], we investigated whether OMS treatment led to mtROS expression in Mabc-infected BMDMs. OMS treatment significantly increased intracellular mtROS levels in BMDMs, and the mtROS levels were increased more in Mabc-infected BMDMs (Figure 7(a)). As mtROS expression is required for inflammatory cytokine production in macrophages [37], we further investigated whether mtROS production was involved in OMS-induced M1-inflammatory cytokine expression in macrophages. Treatment of BMDMs with the mtROS scavenger MitoTEMPO significantly reduced OMS-induced *Tnfα*, *Il12p40*, and *Il6* mRNA levels (Figure 7(b,c,d)) in Mabc-infected macrophages. These data indicated that OMS triggers mtROS expression, leading to upregulated proinflammatory responses in BMDMs during Mabc infection.

NO is critically involved in antimicrobial responses through a synergistic interaction with ROS, which produces a variety of toxic intermediates [38]. Therefore, we investigated whether OMS-mediated anti-Mabc responses depended on mtROS and/or NO production in macrophages. Notably, OMS-mediated antimicrobial responses were significantly reduced by treatment with either the mtROS scavenger MitoTEMPO or iNOS inhibitor L-NMMA (NG-methyl-L-arginine) in Mabc-infected BMDMs (Figure 7(e)). The use of pharmacological inhibitors (MitoTEMPO and L-NMMA) did not affect the cell viability until 48 hr (data not shown). There was no significant difference in intracellular Mabc growth between the MitoTEMPO and L-NMMA treatment conditions (Figure 7(e)). However, the combination of MitoTEMPO and L-NMMA significantly reversed the OMS-induced antimicrobial effects, and led to a higher CFU in the infected BMDMs compared to those treated with MitoTEMPO or L-NMMA alone (Figure 7(e)). Therefore, mtROS and NO production contributed to OMS-induced antimicrobial responses against Mabc infection.

### *OMS and RFB showed synergistic effects* in vitro *and* in vivo

**Figure 8.**
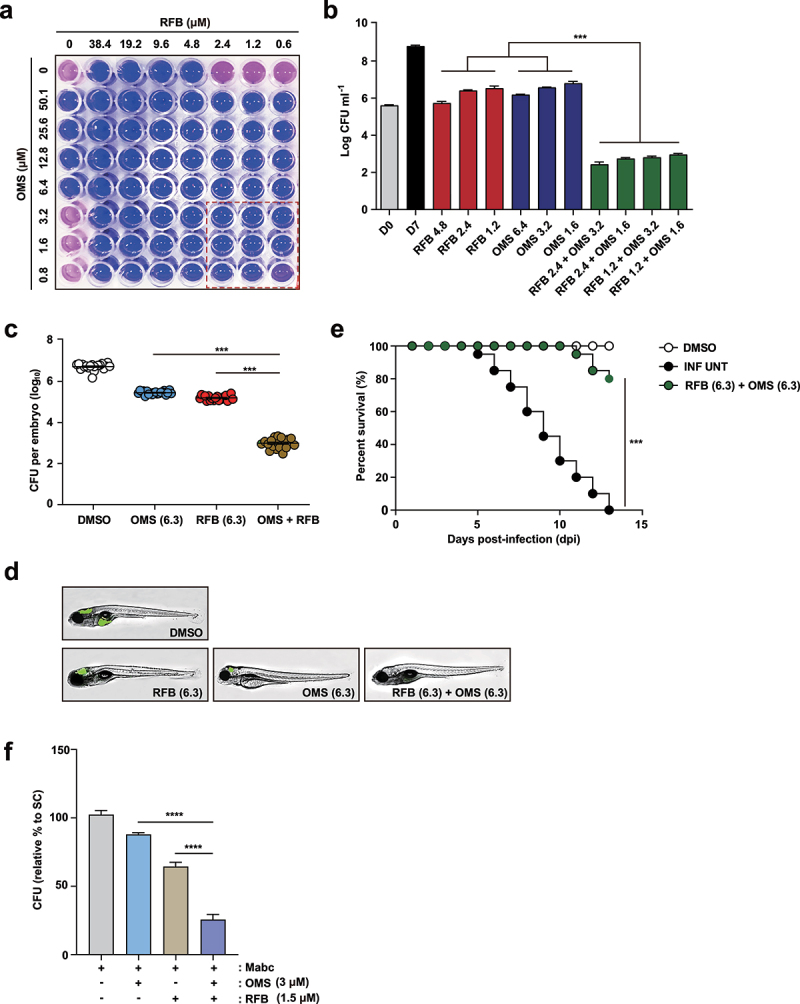
OMS-RFB combination had a synergistic effect *in vitro* and in vivo.(a) Drug interactions were evaluated by chequerboard assay. The MIC_50_ of each drug was in the middle of the concentration range. The white line indicates the MIC_50_ of each compound. (b) Mabc was grown in the presence of different concentrations of OMS alone, or decreasing doses of the OMS-RFB combination. Following 7 days of culture, bacteria were plated on 7H10 agar plates to detect live Mabc. The DMSO-treated bacterial group was also plated on days 0 and 7. One-way ANOVA with Tukey’s multiple comparison test was used to compare means among groups (***p < 0.001). (c) the experiments were performed in triplicate and the results are expressed as the mean log10 CFU per embryo (n = 10 for each condition). (d) a survival curve was plotted for ZF infected with MabcR for 13 days (n = 20, representative of three independent experiments). OMS (6.3 μM) was combined with RFB (6.3 μM). Kaplan-Meier survival curves were constructed using the log-rank (Mantel-Cox) test (***p < 0.001). INF UNT: infected but untreated control. (e) a synergistic effect was also observed, reflected in a reduction in the fluorescence of the mWasabi signal in ZF under a fluorescent microscope. (f) Intracellular survival assay for Mabc (MOI of 1) in BMDMs with or without OMS (3 μM) or RFB (1.5 μM) treatments for 1 day. Statistical significance was calculated using One-way ANOVA with Tukey’s multiple comparison test. Data are presented as mean ±SD. OMS, Ohmyungsamycin A; RFB, rifabutin; CFU, colony forming units; ****, P < 0.0001.

Previously, we used the RFB-anchored anti-Mabc drug combination to identify drug pairs having synergistic effects against Mabc using REMA chequerboard assay Supplementary Figure S4. The OMS-RFB combination had the greatest anti-Mabc activity. OMS concentrations ranging from 0 to 50.1 µM (8 points) were prepared in 96-well plates through 2-fold serial dilution, with an MIC_50_ value (6.4 µM) of OMS in the middle of the concentration range (Figure 8(a)). This OMS concentration was mixed with RFB of various concentrations. The combination of OMS and RFB showed a synergistic antimicrobial effect, with an FIC index of 0.4. Synergy has traditionally been defined as a FIC index ≤0.5 [39]. Thus, the OMS-RFB combination resulted in synergism against Mabc. Half of the OMS MIC_50_ (pink) added to half the RFB MIC_50_ (pink) did not result in a resazurin color change (blue), indicating Mabc growth inhibition. Furthermore, a quarter of OMS MIC_50_ added to a quarter of RFB MIC_50_ also yielded no resazurin color change. To confirm this synergistic effect, a traditional CFU determination assay was also conducted. The combination of 3.2 μM of OMS (half the MIC_50_) and 2.4 µM RFB (half the MIC_50_) exhibited clear growth inhibitory activity (6.4 log10 CFU/mL reduction) compared to untreated DMSO controls on day 7 (Figure 8(b)). Furthermore, the OMS-RFB combination also led to a significant CFU reduction compared to OMS or RFB alone (≥3.3 log10 CFU/mL). Bactericidal activity was defined as a reduction of the total CFU/mL count for the original inoculum of ≥ 3 log10 [40]. Therefore, our data strongly suggest that the OMS-RFB combination is bactericidal against Mabc.

To verify the synergistic effect between OMS and RFB, we used an Mabc-ZF infection and treatment model. As RFB and OMS had a synergistic effect according to the REMA chequerboard assay, their combination was further evaluated in the ZF model. ZF larvae were infected with Mabc and placed in a 96-well plate in the presence of OMS, RFB, or both. Drug concentrations that yielded a CFU reduction of approximately 1 log10 on an agar plate compared to untreated controls were determined (data not shown). OMS and RFB concentrations of 6.3 µM were used for the in vivo combination assay (Figure 8(c)). The synergistic effect was evaluated in three different ways. First, the ZF bacterial burden was estimated by traditional CFU counts. Infected and treated ZF were homogenized, and the number of bacteria was counted on a 7H10 agar plate. As expected, the combination of RFB (6.3 µM) and OMS (6.3 µM) led to significant bacterial CFU reduction (Figure 8(c)). The combination led to a 3.7 log10 reduction compared to untreated DMSO controls 5 days after injection. Second, proliferation of fluorescent Mabc was observed by fluorescence microscopy analysis after OMS-RFB combination treatment. To confirm Mabc proliferation in ZF treated with the OMS-RFB combination, we also infected ZF with a mWasabi GFP expressing the Mabc strain. GFP levels were determined using the ImageXpress® Pico Automated Cell Imaging System. ZF treated with the RFB-OMS combination showed almost no GFP fluorescence compared to those treated with OMS or RFB and control ZF (Figure 8(d)). Finally, we calculated the survival rate of Mabc-infected ZF after treatment with the OMS-RFB combination. As shown in the Kaplan–Meier survival curve, the OMS-RFB combination resulted in a significantly lower mortality rate compared to untreated controls (Figure 8(e)). The OMS-RFB combination led to 18.5% mortality rate at 13 days after treatment. In contrast, the Mabc-infected and untreated negative controls had a 100% mortality rate, while 100% of the non-infected positive group survived after 13 days. These data strongly suggest that the OMS-RFB combination has a synergistic effect in Mabc-infected ZF models *in vivo*.

We further determined whether the combined treatment of BMDMs with RFB and OMS showed a synergistic antimicrobial effect in BMDMs during Mabc infection. As shown in Figure 8(f), we found that the combined treatment of BMDMs with OMS (3 μM) and RFB (1.5 μM) showed a significant increase in antimicrobial activity against Mabc infection than either treatment did. These data collectively indicate that these two drugs have a synergistic effect on the antimicrobial responses during Mabc infection in macrophages and ZF models.

## Discussion

We demonstrated that OMS exerts its antimicrobial effects both directly and via M1-like host protective mechanisms, i.e. upregulation of TNF-α, IL-1β, IL-12B, and iNOS. OMS is a particularly interesting anti-mycobacterial agent, as it can act on both the host and pathogens. Moreover, OMS is a potent inducer of mtROS, which is essential for M1-like proinflammatory response activation in macrophages. Importantly, OMS exhibited robust *in vivo* antimicrobial effects in both immunocompetent and immunocompromised pulmonary Mabc infection mouse models. Granulomatous lesions in the lungs of Mabc-infected mice were significantly attenuated by OMS treatment. This indicates OMS-induced activation of dual pathways governing bactericidal and host-protective responses during Mabc infection.

We previously showed that OMS-A significantly upregulated antimicrobial responses against Mtb infections in macrophages and Drosophila melanogaster [22]. Our study of active OMS derivatives showed that dehydroxy-OMS-A improved antimicrobial responses, and decreased cell toxicity [41]. In addition, a silkworm infection model showed that OMS-A had antimicrobial activity against Mabc [24]. However, it remains unknown whether OMS increases antimicrobial responses in mammalian cells and mouse Mabc pulmonary infection models. In this study, OMS treatment of BMDMs at a safe dose (5 μM) significantly increased antimicrobial effects against Mabc in macrophages. Also, OMS treatment increased *in vivo* anti-Mabc effects in both immunocompetent and immunocompromised mice after intranasal Mabc infection. There is a lack of adequate *in vivo* animal infection models for studying the therapeutic effects of potential antibiotics, and there are no clinically relevant models to assess pathophysiological responses according to the Mabc infection stage [42]. Several studies have demonstrated that Mtb infections in C3HeB/FeJ mice were pathologically similar to human infections [43–46]. In addition, BALB/c mice are more susceptible to mycobacterial infections than C57BL/6 mice, partly due to greater induction of M2 macrophages [47]. Recent studies have suggested that dexamethasone-treated mouse pulmonary Mabc infections closely resemble human Mabc pulmonary disease [48]. Our data demonstrated that OMS treatment had good therapeutic efficacy against Mabc infections in B/c mice treated with dexamethasone (where these mice were more susceptible to Mabc infection). These data strongly suggest that OMS may have therapeutic potential against Mabc pulmonary infections in humans.

In this study, we showed that OMS treatment of Mabc-infected BMDMs significantly augmented M1-like proinflammatory cytokine generation and iNOS expression. NOS2-deficient mice are highly susceptible to Mtb lung infections [49]. In addition, genetic polymorphisms in the NOS2A gene are associated with an increased risk of pulmonary tuberculosis [50], suggesting a critical role for iNOS in antimycobacterial immunity. iNOS-induced NO production limits intracellular Mabc growth [51]. A recent study showed that type-I IFN is required for NO production by macrophages during Mabc infection [52]. NTM-activated M1 macrophage polarization limits the intracellular survival of NTM bacteria [20]. These data, combined with our findings, strongly suggest that OMS-mediated M1 polarization enhances the antimicrobial responses of macrophages during Mabc infection.

We further showed that OMS-induced amplification of mtROS was required for M1-like inflammatory responses during Mabc infection. Mycobacteria, and their individual components, disrupted host mitochondrial dynamics and functions, thereby affecting bioenergetics parameters such as ROS [53]. In addition, mtROS, which are generated by infectious and inflammatory stimuli, are required for proinflammatory cytokine production in macrophages [37,54]. Our data partly accorded with previous findings that mtROS scavenging by MitoTEMPO or cyclosporin A inhibits cytosolic oxidized mtDNA, thus further downregulating type-I IFN and IL-1β production in Mabc-infected macrophages [55]. Furthermore, our data demonstrated that OMS-induced mtROS and NO production contributes to the antimicrobial responses of macrophages. Reactive nitrogen species (RNS) and ROS exert a bactericidal effect in the context of intracellular bacterial infections by reacting with a variety of essential cellular molecules [38,56,57]. The reaction between superoxide (O^·−^) and NO can generate ONOO^·−^ (peroxynitrite), which is responsible for antimicrobial responses in macrophages against bacterial and fungal infections [58,59]. These data collectively suggest that OMS-mediated amplification of mtROS production contributes to proinflammatory responses in Mabc-infected macrophages. Furthermore, OMS-induced mtROS and NO generation may play a critical role in antimicrobial responses against Mabc infections.

We also tested the synergistic effects of OMS against Mabc in vitro, and in the ZF infection-treatment model. Interestingly, OMA combined with RFB was highly synergistic in vitro, with a FIC value of 0.4 (Figure 7(a)). This observation was further validated in a tractable ZF infection/treatment model. ZF share approximately 70% of their genes with humans and have emerged as a valuable genetically tractable model organism for investigating human diseases [60,61]. The Mabc/ZF embryo model has been widely used to assess antibacterial responses in vivo as an alternative host model for mycobacterial infections [62–65]. Importantly, we found that OMS alone reduced the bacterial burden in ZF embryos, and its efficacy was significantly improved by the addition of RFB. Moreover, the RFB-OMS combination significantly inhibited Mabc growth in ZF, thus extending the lifespan of the infected ZF. As Mabc has intrinsic resistance to most of the currently available antibiotics, development of effective treatment regimens against Mabc infections is required. In this context, RFB and its derivatives are promising repurposed antibiotics for Mabc infections [66,67]. Recent studies indicate that RFB is a potent bactericidal drug for both intra- and extra-cellular forms of Mabc bacilli, and has *in vitro* and *in vivo* activity against the smooth and rough Mabc morphotypes (although the drug susceptibility profile is superior for the rough strain) [68]. Based on the urgent need for new therapeutics for Mabc-infected patients with treatment failure [67], our data highlighted the potential of the OMS-RFB combination against Mabc infection. Taken together, our data suggest that OMS is an effective M1-like adjunctive therapeutic against Mabc infections, either alone or in combination with RFB. Future clinical studies are warranted to determine the value of OMS-incorporating regimens for treating highly resistant Mabc infections.

## Supplementary Material

Supplemental Material

## Data Availability

The data that support the findings of this study are available from the corresponding authors, Eun-Kyeong Jo, Jichan Jang, and Dong-Chan Oh upon reasonable request.
