# Supplementary figures and images for: Ohmyungsamycin promotes M1-like inflammatory responses to enhance host defence against Mycobacteroides abscessus infections
Source: Virulence. 2022 Nov 15;13(1):1966–84. doi: 10.1080/21505594.2022.2138009 (PMC9673965; doi:10.1080/21505594.2022.2138009)

**Figure S1**

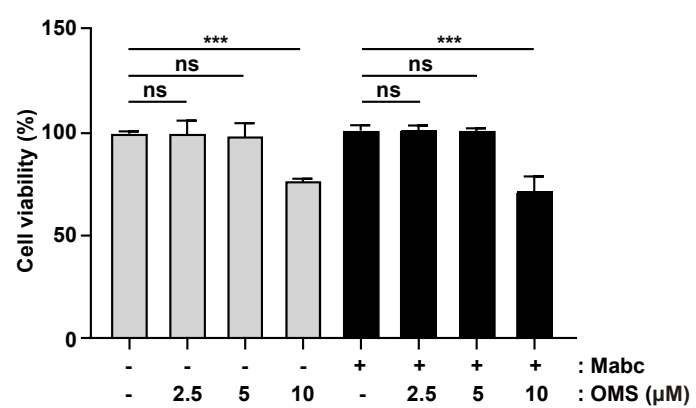

Figure S2

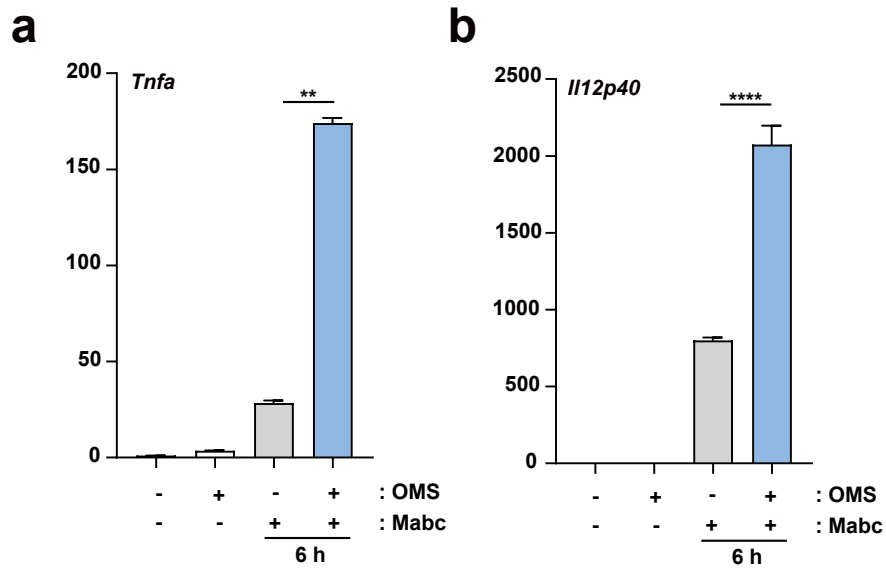

**Figure S3**

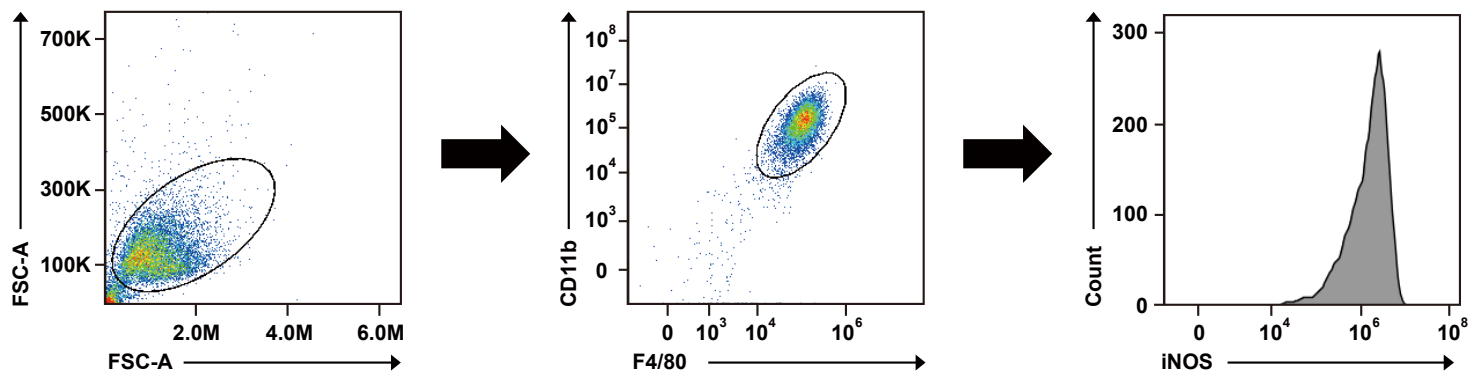

Figure S4

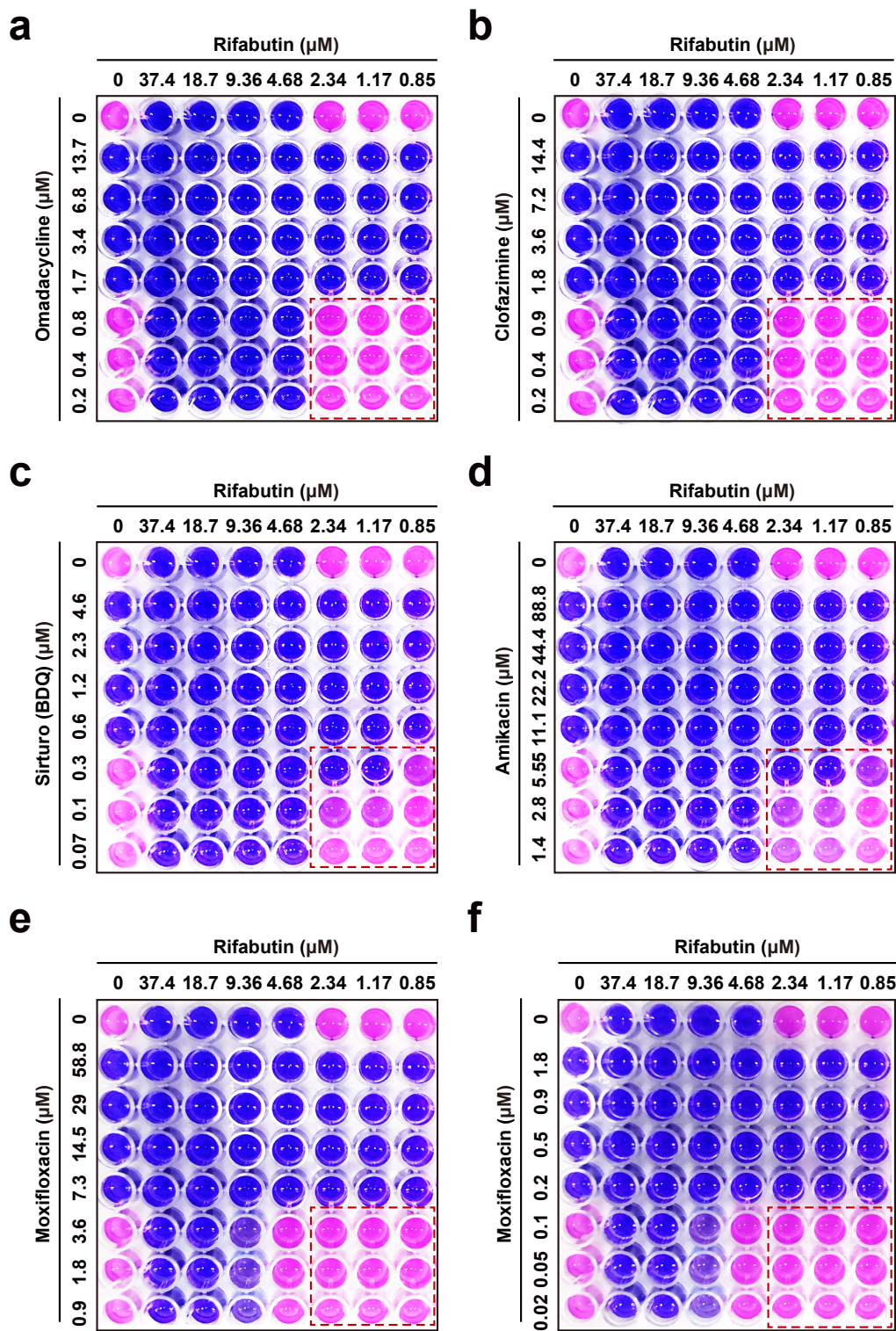

Supplement: Supplemental Material [file KVIR_A_2138009_SM6887.pdf]
